# Lightweight Deep Neural Network Embedded with Stochastic Variational Inference Loss Function for Fast Detection of Human Postures

**DOI:** 10.3390/e25020336

**Published:** 2023-02-11

**Authors:** Feng-Shuo Hsu, Zi-Jun Su, Yamin Kao, Sen-Wei Tsai, Ying-Chao Lin, Po-Hsun Tu, Cihun-Siyong Alex Gong, Chien-Chang Chen

**Affiliations:** 1 Bio-Microsystems Integration Laboratory, Department of Biomedical Sciences and Engineering, National Central University, Taoyuan 320317, Taiwan; 2 Department of Psychiatry, Taichung Tzu Chi Hospital, Buddhist Tzu Chi Medical Foundation, Taichung 427213, Taiwan; 3 Department of Computer Science, College of Computer Science, National Yang Ming Chiao Tung University, Hsinchu 30093, Taiwan; 4 Department of Physical Medicine and Rehabilitation, Taichung Tzu Chi Hospital, Buddhist Tzu Chi Medical Foundation, Taichung 427213, Taiwan; 5 Department of Neurological Institute, Taichung Tzu Chi Hospital, Buddhist Tzu Chi Medical Foundation, Taichung 427213, Taiwan; 6 Department of Neurosurgery, Chang Gung Memorial Hospital, Linkou Branch, Taoyuan 33304, Taiwan; 7 Department of Electrical Engineering, College of Engineering, Chang Gung University, Taoyuan 33302, Taiwan

**Keywords:** bayesian neural networks, gaussian mixture model, human posture identification, integer-arithmetic-only, lightweight neural networks, self-attention, stochastic variational inference

## Abstract

Fusing object detection techniques and stochastic variational inference, we proposed a new scheme for lightweight neural network models, which could simultaneously reduce model sizes and raise the inference speed. This technique was then applied in fast human posture identification. The integer-arithmetic-only algorithm and the feature pyramid network were adopted to reduce the computational complexity in training and to capture features of small objects, respectively. Features of sequential human motion frames (i.e., the centroid coordinates of bounding boxes) were extracted by the self-attention mechanism. With the techniques of Bayesian neural network and stochastic variational inference, human postures could be promptly classified by fast resolving of the Gaussian mixture model for human posture classification. The model took instant centroid features as inputs and indicated possible human postures in the probabilistic maps. Our model had better overall performance than the baseline model ResNet in mean average precision (32.5 vs. 34.6), inference speed (27 vs. 48 milliseconds), and model size (46.2 vs. 227.8 MB). The model could also alert a suspected human falling event about 0.66 s in advance.

## 1. Introduction

Timely detection of human fall events is vital in various care environments. Current technologies of fall detection include wearable devices [1,2], environmental sensing schemes [3], and vision-based methods [3,4,5,6,7,8,9]. The inconvenience of wearing sensors impedes relevant advances [4] and promotes the development of contactless smart sensors. Thus, vision-based methods have become mainstream. Skeleton-based [6,9] and image-based [3,4,5,7,8] posture detections are two primary strategies. Nevertheless, the high-cost apparatus for constructing human skeleton images hinders its development [3,4]. Image-based approaches employing deep neural networks with high structural complexities and computational costs require significant inference time and may jeopardize the detection performance of fall incidents. 

Single-shot multi-box detector (SSD) is a one-stage approach for object predictions using multi-bounding boxes [10,11,12]. Compared to the YOLO series [13,14] or the two-stage method Faster-RCNN [15], SSD has a significantly higher speed of instant inspection. However, the conventional SSD employs VGG16 as its backbone [10], and the architecture still requires extensive model training and prolongs the inference time. Provided the heavy communication loads within the configuration of modern deep convolutional neural networks and unsatisfactory speed, lightweight neural networks emerged and have become the leading technique [16,17]. Among the lightweight neural networks, MobileNet adopts depth- and point-wise separable convolutional layers to decrease computational complexity and parameters [18,19,20,21]. MobileNetV2 adds layers of inverted residuals and linear bottlenecks, for which the feature extraction and data transmission occur in the high- and low-dimensional spaces, respectively [22,23]. With linear bottleneck layers as activations, MobileNetV2 further reduces parameters and computational costs. SqueezeNet [24] and deep compression [25] also open an avenue for model compression by effectively assigning 1 × 1 kernels (a point-wise-like layer structure) to replace the conventional ones. Integrating the architectures of depth-wise separable convolutions and ResNet bottleneck [23], the operation of channel shuffle proposed by ShuffleNet compresses the computational costs while preserving feature information [19]. ShuffleNet V2 [26] further verifies that the reduction in convolutional branches and the replacement of element-wise operations by layer concatenation could significantly raise the computational performance. Distinct from techniques of simplifying neural network architectures or optimizing computational procedures, the quantization training method of integer-arithmetic-only (IAO) algorithm [27,28] provides an alternative to reduce model sizes and accelerate inference by controlling weight bit-widths of convolutional kernels and activation functions. This algorithm also highlights that sophisticated deep convolutional neural networks are impractical baseline architectures because of overusing internal parameters and manually assigning channels in each layer. Under the demand of importing lightweight neural networks, utilizing quantization-aware training [27] in deep neural networks benefits inference performance.

With the combination of a lite SSD network, IAO algorithm, and lightweight neural networks, we established a new framework with much less model structural complexity and better inference capability. To compare the ability of different lightweight neural networks in terms of model size and inference speed, we employed them as backbones of the lite SSD network. In addition, to efficiently reinforce the capability of identifying objects with diverse scales, we embedded the feature pyramid network (FPN) [29] into the lite SSD network to extract local information. Moreover, the self-attention mechanism [30,31] and the residual blocks of ResNet [23] were adopted to process sequential bounding boxes (BBoxs) generated by the lite SSD network for parallelly extracting weighted centroid features of human postures at each time point. These feature maps then became inputs of the variational inference Gaussian mixture model [32] and backpropagation for further classification analysis. Therefore, based on the integrations of these techniques, this framework achieved a fast human posture classification with small model sizes and high inference speed. The key contributions of our study as listed as follows:

- Decreasing model sizes while increasing mean average precisions and inference speeds;
- Incorporating the self-attention mechanism for human posture prediction and data point clustering;
- Using a loss function constructed by Bayesian stochastic variational inference with the distributions rather than the coordinates of data points to reduce the computational complexity significantly and raise tolerance to outliers;
- Providing the probabilistic map to predict falling incidents in a timely manner;
- Validating that the types and observing directions of sensors for data acquisition would not affect the accuracy of the probabilistic map exhibition, i.e., highly compatible with various environments.

In the Materials and Methods section, we describe the datasets, data preprocessing operations, and the compositions of the neural network, including the backbone models and the baseline. We also explain how the proposed framework reduced computational complexity and detected small-size objects. A specific loss function is developed based on the theoretical foundation of Bayesian stochastic variational inference. By incorporating the self-attention mechanism and the backpropagation, the framework updates the loss function’s statistical parameters according to the detected information. In Results and Discussion section, the performance comparison of the backbone models for fast object detection and prediction is exhibited. The model with the best performance is selected for our framework. The generalization capabilities of the loss function under different devices and environments are also demonstrated. Finally, we propose a probabilistic map for the prediction of human postures. In Conclusion section, we summarize the achievements of the proposed framework and the future recommendations.

## 2. Materials and Methods

**Figure 1:**
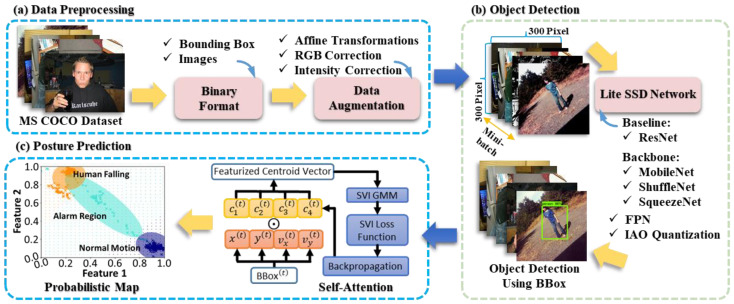
The proposed framework, including (**a**) data preprocessing using the MS COCO dataset for training and the ImageNet [34] for data augmentation, (**b**) establishment of a lite SSD network for fast human detection, and (**c**) the integration of statistic learning and self-attention mechanism for human posture prediction and clustering.

Figure 1 illustrates the proposed framework and experimental procedures. For data preprocessing in part (a), we adopted the MS COCO dataset [33] and applied binary transform and data augmentation, including affine transformations, RGB correction, and intensity correction. For object detection in part (b), the input images were standardized before being sent into the lite SSD network with FPN and IAO using MobileNet, ShuffleNet, or SqueezeNet as the backbone. ResNet was also used as the baseline for comparison. For posture prediction in part (c), the locations and speeds of the extracted BBox centroids were the input features of the self-attention block. The feature vectors carrying centroids and clustering properties then delineated in the probabilistic map of human postures estimated by the Bayesian-based model.

### 2.1. Establishment of Binary Format and Data Augmentation

The MS COCO, Pascal VOC2012 [35], and ImageNet [34] are open-access datasets extensively utilized for object detection. Among these, the MS COCO has the most detectable objects (i.e., BBox numbers) and the most balanced object sizes (equal portions of small, middle, and large objects). Hence, the MS COCO database is more in line with the daily environment and thus can achieve a better effect on deep learning model training on object recognition. We first transformed the labels and images into a binary data format to facilitate the reduction in loading time and the efficiency of parallel operations. Then we took the geometric (affine) transformations to avoid overfitting caused by the uneven distributions of image sizes and increase image content information. The affine transformations employed to the BBoxs and the images included rotations, flipping, random cutting, and deformations. We also randomly adjusted color brightness, saturation, hue, and contrast to simulate different real-world environments. To avoid overexposure or underexposure of the input images, we applied the mean values of RGB channels of the ImageNet dataset to whiten all input images.

### 2.2. Design of Lite SSD Network and Model Selection

As shown in the performance of lightweight neural networks presented by Lin et al. [29], the shallower the neural layers, the better detection of small objects, but the weaker information on locations. Conventional networks adopt featurized image pyramids that utilize multi-scale-fused features in training procedures to address this issue; however, it increases the inference time. Thus, we employed FPN to locate small objects in the shallow layers efficiently. Structurally, FPN extracts meaningful features with deep convolutional layers and then grasps better position information through up-sampling. It then fuses feature maps with the same size to preserve the original recognition scales. Meanwhile, to avoid the aliasing effect occurring in up-sampling procedures, we added a convolutional layer after the feature map fusing in the lite SSD network.

To pursue real-time object detection and implementation in lightweight neural networks, we considered the data-loading speed and storage size as vital factors for framework optimization. It is because the structural complexity of a neural network affects training efficiency and inference speed. In our experiments, we found that using FLOPs (floating-point operations per second) to evaluate the structural complexity might not reflect the actual inference speed. Reference [22] evidences the results. Thus, in addition to model size and inference speed, we adopted mean average precision (mAP) estimation [13] to evaluate the robustness of the backbones and the baseline. We also observed the performance of quantization-aware training of the IAO algorithm in this stage.

### 2.3. Theoretical Foundation of Stochastic Variational Inference Gaussian Mixture Model with Self-Attention Mechanism

As illustrated in part (c) of Figure 1, the self-attention mechanism extracts instant centroid locations (i.e., $x^{\left(t\right)}$ and $y^{\left(t\right)}$) and speeds (i.e., $v_{x}^{\left(t\right)}$ and $v_{y}^{\left(t\right)}$) at the time t from the detected ${\mathrm{BBox}}^{\left(t\right)}$. The parameters $c_{i}^{\left(t\right)}$, i=1, 2, 3, 4 are the corresponding weights to the instant centroid features. The symbol ⨀ represents the operation of the Hadamard product between the vectors. The preprocessed sequential data generated vectors containing time and position information between ${\mathrm{BBox}}^{\left(t\right)}$ and ${\mathrm{BBox}}^{\left(t+20\right)}$, which became feature vectors for cluster analysis. The loss function derived from stochastic variational inference (SVI) and the backpropagation train the parameters used in the self-attention block and statistical distributions. Then, the results combined with the Gaussian mixture model (GMM) present the possible states of human motions in the probabilistic map.

The Bayesian neural network uses a set of variational distributions q(z) to approximate the posterior distributions p(z|s). The logarithmic probability density function (PDF) of a sample lnp(s) can often be expressed as the linear combination of evidence lower bound (ELBO) and the Kullback–Leibler divergence (KLD) [36,37,38]:

(1) $$\ln p\left(s\right)=\int_{z}^{}q\left(z\right)\ln\left(\frac{p\left(s,z\right)}{q\left(z\right)}\right)dz+\mathrm{KLD}\left(q\left(z\right)||p\left(z|s\right)\right),$$

where s and z represent sample data vectors and latent variable vectors, respectively. The goal of the variational inference is to achieve the maximization of ELBO and the minimization of KLD simultaneously through variational calculation under the condition that lnp(s) is a constant. Minimizing KLD means that the variational distribution should be similar to the posterior distribution, so that we only need to consider maximizing the ELBO under this constraint. The convenient way to find the extremum of the ELBO is to introduce the mean-field theory into the variational distribution [38]. However, this technique relies on taking all samples to update the variational distribution, computational complexity would arise. When the posterior distribution becomes more complicated, it also needs more iterations of variational distributions. All these operations lead to high computational costs and structural uncertainty under the circumstances of large datasets and complicated posterior forms [36,37].

We proposed a new technique based on the structure of stochastic variational inference to conquer these problems. It can resolve the issue of complicated posterior form and reduce computational costs through mini-batches. It also made the variational distribution approach the posterior distribution by maximizing the ELBO and adapting the ELBO into a tractable PDF:

(2) $$\begin{array}{l} \mathrm{ELBO}=\int_{z}^{}q\left(z\right)\ln\left(\frac{p\left(s,z\right)}{q\left(z\right)}\right)dz\\ \;=\int_{z}^{}q\left(z\right)\ln p\left(s|z\right)dz+\int_{z}^{}q\left(z\right)\ln p\left(z\right)dz-\int_{z}^{}q\left(z\right)\ln q\left(z\right)dz\\ \;\approx \frac{1}{L}\overset{L}{\underset{l=1}{\sum }}\ln p\left(s|z\right)-\mathrm{KLD}\left(q\left(z\right)||p\left(z\right)\right). \end{array}$$

The terms p(s|z) and p(z) are variational likelihood and prior distribution, respectively. Notice that we used the discrete sampling form to replace the Monte Carlo integration in the first term of Equation (2). The parameter L is the sampling size. To integrate this result into the deep learning structure, we further modified the ELBO in Equation (2) as a loss function ℒ(s,z) so that the backpropagation could sequentially update the parameters in the self-attention block and the GMM:

(3) $$ℒ\left(s,z\right)=-\left[\frac{1}{L}\overset{L}{\underset{l=1}{\sum }}\ln p\left(s|z\right)-\mathrm{KLD}\left(q\left(z\right)||p\left(z\right)\right)\right].$$

Equations (2) and (3) jointly show that ELBO is equivalent to the linear combination of variational likelihoods and the KLD is constructed by variational distributions and sample priors. Equation (3) implies that when the variational distribution q(z) and prior distribution p(z) gradually become similar during training, the KLD would also approach zero. Then, the logarithmic variational likelihood, the first term of Equation (3), would reach its maximum value due to obtaining the corresponding distributions of latent variables inputs z. Since the KLD in Equation (3) is always positive, the logarithmic variational likelihood can be treated as the lower bound of the loss function. This equation is tractable and has a predictable lower bound. The belonging parameter distributions also can be updated in the training procedures. Thus, these elegant mathematical properties make it suitable to be a loss function. In other words, the loss function established from the ELBO in this study allows us to fuse the technique of backpropagation of deep neural networks with the statistical learning models for more complex analyses.

The prior and variational likelihood distributions were all Gaussian in the study. The relevant initial statistical parameters of the prior distribution p(z) were the mean value $\mu_{prior,\;k}\sim Normal\left(0,1\right)$, the inverse covariance matrix $\Sigma_{prior,\;k}^{-1}\sim Wishart\left(3,\;I_{K}/3\right)$, and the cluster weight $\alpha_{prior,k}\sim Dirchlet\left(2K,\;2K\right)$. Then, the parameters of the variational likelihood distribution p(s|z) were $\mu_{var,\;k}\sim Normal\left(N_{1},N_{2}\right)$, $\Sigma_{var,\;k}^{-1}\sim Wishart\left(W_{1},\;W_{2}\right)$, and $\alpha_{k}\sim Dirchlet\left(D,\;D\right)$. The backpropagation updated the parameter vectors in these distributions, namely $N_{1}$, $N_{2}$, $W_{1}$, $W_{2}$, and D. The factor k was the index of cluster number K, and was assumed to be 2 or 3 in the SVI GMM training. Therefore, the variational likelihood has the form:

(4) $$p\left(s|z\right)=GMM\sim \overset{K=2,3}{\underset{k=1}{\sum }}\alpha_{k}Normal\left(s|\mu_{var,\;k},\Sigma_{var,\;k}\right).$$

Please note that the proposed framework governed the training procedures and updated the statistical parameters of kth prior distribution p(z) sequentially through mini-batches. The collected sequential data point distributions gradually fit the mean value $\mu_{prior,\;k}$, covariance matrix $\Sigma_{prior,\;k}$, and the cluster weight $\alpha_{prior,k}$ of the kth prior distribution p(z) in the training procedures. Then, those optimized statistical parameters from prior distributions p(z) consisted of and updated the parameters of the variational likelihood distribution p(s|z). Since this technique used only the distributions instead of the original position of data points, it reduced the computational complexity significantly and raised outlier tolerance. Not only could we provide the corresponding probabilistic map without losing the inference performance, but we could also estimate the posterior distributions p(z|s) by employing the outcome from Equation (4) directly:

(5) $$p\left(z|s\right)=\frac{\alpha_{z}Normal\left(s|\mu_{z,\;k},\Sigma_{z,\;k}\right)}{\sum_{k=1}^{K=2,3}\alpha_{k}Normal\left(s|\mu_{var,\;k},\Sigma_{var,\;k}\right)}.$$

the parameters $\alpha_{z}$, $\mu_{z,k}$, and $\Sigma_{z,\;k}$ are the cluster weight, mean value, and covariance matrix of the data cluster constructed by the variational distribution q(z), respectively.

## 3. Results and Discussions

### 3.1. Performance Comparison of the Object Detection Models

To fairly compare and inspect the capability of the proposed framework, we employed MoblieNet, ShuffleNet, and SqueezeNet as backbones of our lite SSD networks, in which FPN and IAO algorithms were incorporated to enhance small object detection, reduce model sizes, and raise the inference speed. We also adopted the ResNet as the baseline model for performance comparison. The backbone models were the main techniques employed for object detection, so their intrinsic performance indicated the general effectiveness. Table 1 summarizes the comparison results of these backbones incorporated with FPN and the IAO algorithm. The overall performance of mAP, inference speed, and model size reflected their potential of being the backbone model in the proposed framework.

ResNet, as the baseline, generated a fair mAP of 24.6 but a relatively slow speed of 48 mSec and a large model size of 227.8 MB. MobileNetV1 and MobileNetV2 had similar mAPs (32.5 and 33.6) and inference speeds (27 and 25 mSec), but MobileNetV1 had a much smaller model size than that of MobileNetV2 (46.2 vs. 140.2 MB). Because ShuffleNet divided different feature map channels into different groups and operated convolutions separately, general *For* loop iterations and single GPU parallel iterations were used to inspect its performance. Although *For* loop iterations and parallel convolution operations had faster inference speeds (20 and 16 mSec), their model sizes were not small enough. ShuffleNet did not effectively support TensorFlow Lite (TFlite) [26]; therefore, incorporating IAO using TFlite resulted in a poor inference speed. SqueezeNet had an extremely fast speed of 10 mSec and a tiny model size of 16.2 MB, but the worst mAP of 16.5. With the best overall performance, MobileNetV1 was selected as the backbone of the lite SSD network. The results listed in Table 1 also validate FPN’s contribution to improving model accuracy and IAO’s ability to accelerate inference speed.

### 3.2. Object Tracking and Human Posture Classification

**Figure 2:**
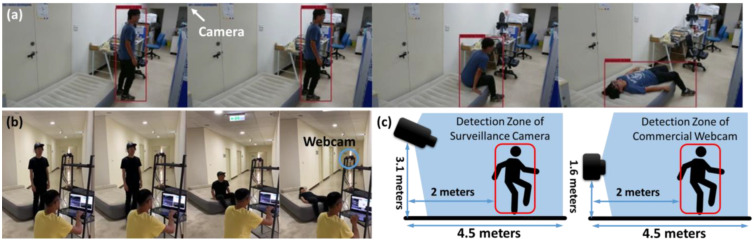
The demonstration of data acquisition from (**a**) a surveillance camera and (**b**) a commercial webcam. (**b**) The scene of inference performance testing, and thus there were no BBoxs on the subject. (**c**) The critical dimensions of the experimental environments using a surveillance camera and a commercial webcam.

There were 15 healthy subjects with a mean height of 158.6 ± 14.3 cm in our study. As shown in Figure 2, in-house-made 60 FPS (frame per second) videos were collected from each subject using a commercial webcam and a surveillance camera. We employed only low-resolution images in this study to achieve fast object detection. The two apparatuses were set at different heights to simulate different data acquisition environments with the camera at 3.1 m and the webcam at 1.6 m. The two data sources helped to validate whether the SVI GMM could map different data types to the same probabilistic map. Subjects were asked to rotate in place for 30 s to imitate the dizzy situation before falling onto the air mattress with consciousness. The protocol matched the requirement of [3].

**Figure 3:**
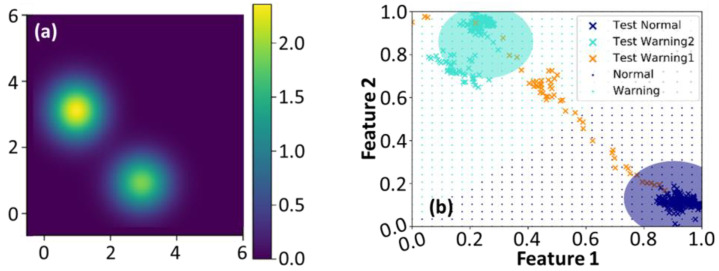
(**a**,**b**) The variational likelihood map and the normalized map in the feature space estimated by Equations (4) and (5) with K=2 and diagonal covariance of GMM, respectively.

**Figure 4:**
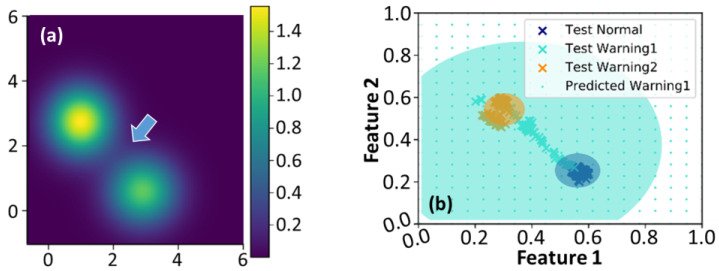
(**a**,**b**) The variational likelihood map and the normalized map in the feature space estimated by Equations (4) and (5) using K=3 and diagonal covariance of GMM, respectively. The arrow depicted in (**a**) indicates that the boundaries of these two groups become blurred.

To further explore the possible reduction in computational complexity of SVI GMM, the accuracies of classified results were analyzed by employing the diagonal and full covariances of the GMM. We initially assigned K=2 in Equations (4) and (5) and used diagonal covariance to simplify the computational cost. Figure 3 demonstrates the corresponding variational likelihood map in (a) and the normalized feature map [39] in (b). The green and blue dot grids in Figure 3b represent the warning and normal regions, respectively. The cross markers represent the actual data points classified by the SVI GMM. Only two groups are delineated in Figure 3a,b since K equals 2; however, unclassified data points appear between those clusters. It implies that this dataset should have more than two groups [3,39]. Figure 4 shows the corresponding maps estimated by Equations (4) and (5) with K=3 and diagonal covariance. The utilization of the oversimplified GMM covariance caused bizarre classified results. The group consisted of the unclassified data points, as those orange cross markers depicted in Figure 3b eventually dominated the classification. It also resulted in blurred group boundaries and reduced the maximum value in the variational likelihood map. In other words, utilizing the diagonal covariance of GMM with K=2 or K=3 would increase the uncertainty of data classification.

**Figure 5:**
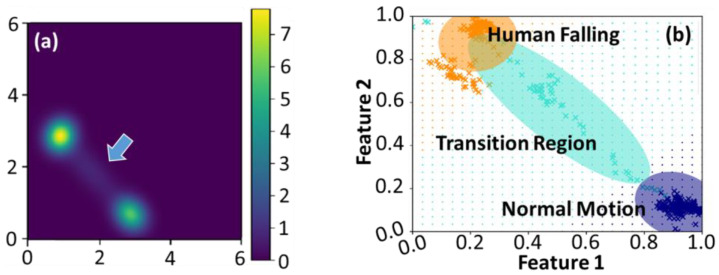
(**a**) The variational likelihood map estimated by Equation (4) and (**b**) a normalized map in the feature space. The colored regions in (**b**) exhibit the human posture classification. The arrow depicted in (**a**) implies there is a transition region between these two groups.

To reduce the classification uncertainty and reinforce the likelihood estimation, we eventually employed full covariance of the GMM and K=3 in the proposed framework for human posture classification. Figure 5 illustrates the variational likelihood map and the corresponding normalized map in the feature space after the SVI GMM training. The intensity of the groups in the likelihood map became more concentrated. Predicted by the training datasets, the blue, green, and orange dot grids shown in Figure 5b indicate the regions of normal motion, transition warning, and falling, respectively. The cross markers represent the actual data points of normal motions, transition motions, and falling, respectively. Then the blue, green, and orange ellipse regions are the Eigen-matrices of the covariance of likelihoods corresponding to Figure 5a. These Eigen-matrices reflect the uncertainty of data variations and provide the visualization of the discriminant distributions. When a falling event occurs, the data points of posture features would sequentially distribute from the normal motion region through the transition region and then reach the falling regions. This procedure took about 0.66 s and underwent 40 extracted BBox centroid points. Table 2 lists the quantitative analysis of data point classification under the proposed framework. Table 3 lists the performance comparison between state-of-the-art techniques and the proposed framework. It should be emphasized that only the proposed framework inferred fast enough to generate alarm warnings before a human falling event happens. 

**Figure 6:**
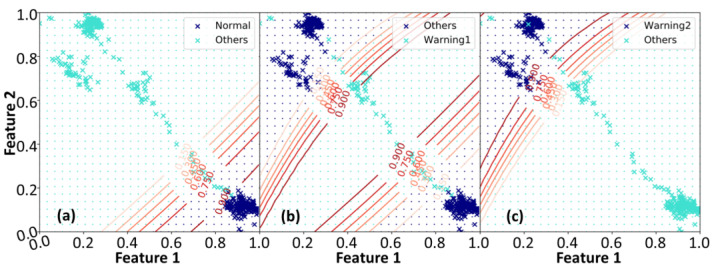
The demonstrations of the probabilistic maps constructed by the SVI GMM. (**a**) The distinct distributions of normal motion (the blue cross markers) and other situations (the green cross markers). (**b**) When the detected centroid points, as depicted by the green cross markers, were in the transition region, the proposed framework would generate the first alarm warnings. (**c**) When the detected centroid points, as depicted by the blue cross markers, were in the falling region, the proposed framework would generate second alarm warnings.

Figure 6 exhibits the probabilistic maps established using the SVI GMM. This method mapped the BBox centroid points into three distinct predictive situations. Then, the SVI GMM endowed these points with their corresponding probability values. The centroid points were in the normal region of Figure 6a when the subject walked or stood normally. When the detected centroid points moved into the transition region (Warning1) of Figure 6b and migrated toward the falling region (Warning2) of Figure 6c, the system would generate alarm warnings immediately.

## 4. Conclusions

This article provides a new framework for lightweight deep neural network modeling, and it meets the demand for fast classifying of human posture images and subsequent warnings. This framework simultaneously achieves high mean average accuracy, high inference speed, and small model size of object detection tasks. The method uses a commercial webcam and a surveillance camera for data acquisition. It matches the requirement of contactless human posture detection. This method has a form of lite SSD network embedded with quantization-aware training and a self-attention mechanism, and thus it can reduce model sizes and raise the inference speed. The framework can fuse the information from images and corresponding sequential signals obtained from bounding boxes. The proposed method merges the techniques of statistical learning into deep learning. Hence, the trained parameters own their statistical meanings. The classified results of images and corresponding sequential signals could be mapped onto probabilistic maps directly. Therefore, this lightweight structure could quickly estimate the probability of human postures and generate alarms once the corresponding data points move into the warning regions. This method connects the loss function with the technique of stochastic variational inference. Thus, it endows the notions of probability to the classification inference. Since the framework has a superior achievement on inference speed and model size, it is a strong candidate for low-cost applications of edge computing and embedded systems. Furthermore, the framework can be the baseline for developing tiny machine learning (TinyML) techniques or other lite structural platforms. Therefore, we anticipate this framework can benefit the progress of contactless smart sensing and detection in biomedical AIoT developments.

## Figures and Tables

**Table 1:** The performance comparison of backbone models.

Backbone	FPN	IAO	mAP	Inference Speed (mSec)	Model Size (MB)
ResNet			24.6	48	227.8
MobileNetV1			21.2	15	74.1
	✓ ^a^		33.1	28	132.9
		✓	20.6	13	28.2
	✓	✓	32.5	27	46.2
MobileNetV2			23.0	17	174.9
	✓		35.6	29	320.1
		✓	22.7	12	94.9
	✓	✓	33.6	25	140.2
ShuffleNet V1 (Group = 4)			20.7	20	77.6 ^b^
			20.7	16	77.6 ^c^
		✓	20.3	-- ^e^	34.6 ^d^
ShuffleNet V2			21.9	18	55.1
SqueezeNet			16.5	10	16.2

^a^ The method was employed. ^b^ Using *For* loop iterations. ^c^ Using GPU parallel iterations. ^d^ Using TFlite framework. ^e^ Very poor inference speed.

**Table 2:** The quantitative results of the proposed framework.

**True Positive**	**True Negative**	**False Position**	**False Negative**
26,857	11,472	3015	1208

**Table 3:** The performance comparison between state-of-the-art techniques and the framework.

Source	Apparatus	Method	Accuracy	Alarm Timing ^1^
Ref. [3]	Sensor Fusion	Machine Learning	0.90	+0.7 s
Ref. [4]	Vision-based Method	SpeedyAI, Inc.	0.89	+10 s
Ref. [6]	Vision-based Method	CNNs	0.98	-- ^2^
Ref. [7]	--	3D CNNs	0.99	--
This work	Vision-based Method	Lite SSD	0.90	−0.66 s

^1^ + and −: The alarm will occur after and before the human falling events, respectively. ^2^ --: Not available.

## Data Availability

Data sharing not applicable.
